# Women in Need—The Nature of Social Support in an Online Community

**DOI:** 10.3390/bs13090726

**Published:** 2023-08-30

**Authors:** Vanja Ida Erčulj, Tinkara Pavšič Mrevlje

**Affiliations:** Faculty of Criminal Justice and Security, University of Maribor, 1000 Ljubljana, Slovenia; vanja.erculj@um.si

**Keywords:** online support, social support, violence against women

## Abstract

Some women, especially victims of violence, seek support in online groups. The objective of this research was to investigate the nature of the social support women receive in such online communities. For this purpose, all the posts from a large online support community group for women in Slovenia, Women in Need, from 2002 to the end of 2020 were retrieved and analyzed manually as well as by using a text-mining approach. The results show that women in the investigated community mostly seek informational support, spend little time actively contributing to online discussions, and rarely become engaged members of supportive groups within the focal online space. Some recommendations on how to improve the functionality of online social support sites are provided.

## 1. Introduction

Violence against women encompasses any act of force that seriously threatens a woman’s life, body, psychological integrity, or freedom, the most prevalent being the abuse of women by intimate male partners [1]. According to the World Health Organization (WHO), one in three women is a victim of intimate partner violence (IPV) or another type of violence in her lifetime [2]. The classification of IPV includes physical, sexual, and/or psychological abuse [3]. The European Union Agency for Fundamental Rights (FRA) [4] further elaborates that IPV (and other types of violence against women) is underreported to the police or other institutions providing help to women and victims of crime. A systematic review of studies exploring the consequences of reporting violence to informal or formal sources showed that the negative social reactions (e.g., blame and disbelief) women might receive after disclosure can lead to more avoidance coping mechanisms, lower perceived control over the situation, and self-blame [5]. This systematic review analyzed 30 studies on correlates of social reactions to disclosure of sexual assault or IPV and showed that negative social reactions tend to be present to a higher extent when a victim is less educated, bisexual, reports an extensive trauma history, alcohol use, or having a long-term abusive relationship with the offender. In contrast, positive reactions are present to a greater extent when women seek support from informal sources, such as friends, family members, or neighbors. However, some studies report that, while women often tell someone about the abuse they are suffering, their friends do not play an important role in helping them to exit the violent relationship [6]; moreover, 40% of such women received no help, possibly because of a lack of skills and knowledge with regard to how to handle such situations [7].

Social support typically refers to support individuals have received from others (i.e., received support) but also to the degree to which they perceive that others are available as a support resource (i.e., perceived support) [8]. Research of social support as a potential protective factor shows mixed results; however, the majority of studies show that battered women have low perceived social support [9]. More specifically, women who receive little social support and live in disadvantaged neighborhoods are at the highest risk of partner violence [10]. Interestingly, social belonging is associated with an increased probability of informal support seeking but not with seeking help from any other source [11]. Additionally, looking for help from various sources is needed, as there is a constant overlap of information needs, since victims need to deal with more than one issue at the same time due to problems related to money, legal help, social support, health, accommodation, and so on [12].

Another important obstacle to seeking help can be of an emotional nature, as shame and even guilt may deter an abused woman from acting on the decision to leave a violent relationship [13]. Indeed, a review of IPV online interventions shows that guilt, shame, humiliation, and stigma often deter women from seeking face-to-face help [14], which might make the online context a good solution, as a woman can look for information anonymously and at her own pace. Moreover, it might be a form of social support that their violent partner is not able to completely control.

Another advantage of online support groups is that they consist of members with similar experiences. Such similarities can increase the understanding between support seekers and providers and result in social support that is better able to meet the needs and goals of the former. This is a crucial element of high-quality social support, as argued by the optimal matching model [15]. This model states that the effects of social support are enhanced when it meets the needs for support expressed by the support seeker. As online social support is readily and constantly available and includes a greater number of possible support providers, it can more easily meet the needs of the support seeker. Furthermore, it enables asynchronous exchanges and the provision of novel information by a large number of members. The easy accessibility and constant presence of online groups are crucial aspects of the high-quality social support they can offer [8]. The level of social support a woman receives is highly related to the frequency of their interactions and the amount of time they spend online. More proactive members can receive a greater amount of social support than their less engaged peers. An investigation of the online social support provided to victims of IPV showed that many elements of face-to-face support can also be delivered effectively online [16]. However, online interventions are most effective in encouraging women to leave their abusive partners but deal with what follows afterwards to a much lesser extent [14]. As such, women often do not receive enough information on how best to act after they leave an abusive relationship.

Regarding the type of social support that women receive online, a Chinese study of Twitter messages posted by female victims of IPV showed that emotional support is more common than informational support [17], with emotional support including offering good wishes, encouragement, and empathy, while informational support consisted of providing explanations, sharing personal experiences, and giving information on immediate and long-term actions. However, this later point somewhat contradicts the findings of a review of studies from Canada, New Zealand, and the USA by Rempel et al. [14], which stated that online support fails to focus on long-term actions (i.e., actions after leaving an abusive partner). An analysis of five American Q&A sites [18] revealed that women mainly ask general questions about IPV and seek advice on financial, health, and legal issues. They also ask binary questions about choosing one of two possible options or seeking explanations about “the nature of the abusive relationship, the reasoning behind actions, the process for task completion, and the potential outcomes of events” [18]. On the Q&A sites, there seems to be a greater extent of informational support seeking and provision, while emotional support comes in the form of encouragement. Other types of social support have rarely been found or identified in research studies analyzing the content of online discussions. Some women in a Chinese study sought instrumental support, such as free temporary lodging, but such posts were rare [17]. Another study [19] revealed that the primary motives for visiting such an online group by victims of sexual violence were to find a supportive community, seek advice, and tell their own stories. In another study [20], rich storytelling was found to be one of the techniques for obtaining emotional support. Among all types of social support that are available, emotional support seems to be the most beneficial for the psychological and physical health of the support seeker [8,21]. With regard to the social reactions to the disclosures made by victims of violence, some evidence exists that these are in fact positive in an online setting. For example, a study of online communication about sexual violence on Twitter showed very positive reactions to disclosures, such as the provision of emotional and informational support to the survivor [22].

Seeking and accepting help and support is a complex process that is affected not only by the way in which it is requested or offered. As mentioned earlier, social support is linked to the victim’s social functioning per se and, more often than not, the victim’s functioning in this area is limited. In fact, any past abuse (physical, sexual, or emotional) is significantly associated with impaired social functioning [23]. More specifically, women with recent IPV have lower social functioning scores (e.g., not trusting people in the community) [24], and a pattern of cautiousness and insecurity towards others has been recognized in women in physically violent relationships [25]. In addition, even though the victims did not feel their isolation was forced on them by their partners, Rose and Campbell [25] wonder if the forced isolation was internalized into the women’s sense of self.

The findings suggest that online social support for female victims of violence could have beneficial effects on their intention to leave the abusive relationship and seek help, but it depends to a great extent on the nature of the support these women receive and, thus, investigating this is the main aim of the present study. The research questions that we addressed were as follows:

1. What are the main motives for women to participate in online support group discussions?
2. How many starting posts describe violence and who is the perpetrator?
3. What are the main topics of discussion?
4. What is the nature of the online social support that is provided?

## 2. Materials and Methods

### 2.1. Procedure

All the posts between 2 February 2002 and 20 November 2020 from the first online support communities for women in Slovenia, Women in Need (Ženske v stiski) on the website med.over.net, were retrieved and analyzed. The main purpose of the forum is to help women that need advice or would like to share their experience. Psychologists and social workers who work with female victims of domestic violence on a day-to-day basis moderate the discussions in this community. The user needs an account to engage in the forum to provide freedom of speech and prevent hate speech. There is no age requirement.

Overall, there were 1455 threads and 6909 posts from 4268 unique users. The data were provided by med.over.net and included information about the thread ID, thread title, username, thread date, post ID, post title, post text, and post date. There was no information by which a user’s real identity could be identified. A manual inspection of 600 randomly selected posts’ starting threads showed the users were mainly (97%) women (the Slovenian language permits the gender identity of the author to be revealed, in particular by the use of certain suffixes). As the male participants were rare and felt compelled to contribute to the discussion of the clearly stated female forum, their posts were not excluded from the analysis. Due to the rarity of posts written by males, the inclusion did not in any sense influence the validity of the research findings.

### 2.2. Analysis

The random sample of 600 starting posts was analyzed manually by two independent coders to annotate whether the post described violence and who the perpetrator was. The agreement between coders measured by Cohen’s kappa was 0.80 (*p* < 0.001), which indicates high inter-rater agreement. The discrepancies between the coders were examined and resolved by a third, independent coder. Additionally, the coders annotated the main motives for visiting the online community. The codes were provided beforehand, based on the results of O’Neill [19], which identified three main motives for visiting an online community for victims of sexual violence, namely seeking a supportive community, seeking advice, and storytelling. During the inspection of the starting posts, O’Neill’s coding scheme [19] proved to be insufficient. Therefore, the initial codes were expanded into seeking informational support, seeking emotional support, seeking instrumental (tangible) support, and storytelling. We used Cutrona and Suhr’s definition of each type of social support [26]. Informational support is defined as providing knowledge or facts, such as advice or feedback on actions, while emotional support is defined as the evaluation and acknowledgment of an individual’s feelings or providing comfort, empathy, sympathy, and encouragement. Instrumental support includes providing any goods and services needed to the support seeker. The agreement between coders, as measured by Cohen’s K, was 0.76 (*p* < 0.001), which signifies substantial agreement between coders, and, overall, the two coders assigned the same codes to 94% of posts. All discrepancies were discussed and further examined by a third, independent coder until the final decision and consensus was reached. 

Before applying text mining methods for automatic analysis of the posts, the text was preprocessed. Upper-case words were changed to lower-case words. Stop words (e.g., of, I, we, you, with, is), punctuation, numbers, and words with an overall frequency of less than two were removed from the text. Tokenization (division of the text into units such as words and punctuation marks) and lemmatization (changing different forms of the same word such as “give” and “gives” into a single word) were applied using an online text analyzer [27,28]. 

Automatic content analysis, namely topic modelling using the Latent Dirichlet Allocation (LDA) method, was used to find topics of discussion in all the retrieved posts. The most probable topic of each post was calculated. Ten topics were identified, as this resulted in the clearest topic themes. The 10 most probable keywords per topic were identified, and 100 randomly selected posts from each topic were selected and manually inspected for the content to calculate the homogeneity of the content of the posts in each topic. The share of posts mirroring the topic content along with the 95% confidence interval was calculated for each topic. The share of homogenous posts per topic with regard to content ranged from 80 to 99%, with the lowest lower bound of the 95% confidence interval per topic being 71%. The repetition of the LDA with different random starting assignments of topics to posts and words to topics resulted in a stable solution with regard to the content. 

Network analysis was used to assess the co-operation between members of the online community. Members were represented by vertices, and edges between the members existed if these members co-operated (exchanged opinions) in at least two threads. The analysis included the removal of the vertices with a degree lower than 1 and identification of connected components. By doing this, those groups of participants who co-operated more often were identified.

The nature of the social support offered was further inspected by calculating the number of posts per thread, number of users with each number of posts, and number of users in consecutive years.

The analysis was performed by using the program R (libraries XML, RCurl, and LDA), version 4.1.1, and the program Pajek, version 5.14.

## 3. Results

### 3.1. Description of Violence in the Starting Posts

**Figure 1:**
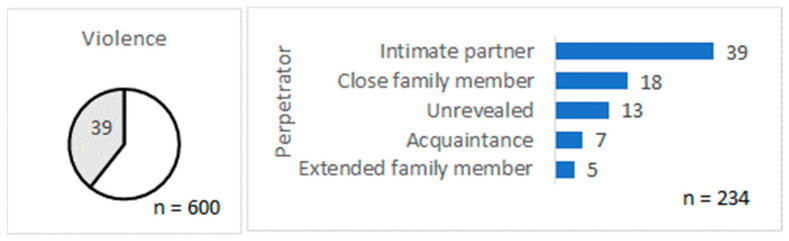
The share of starting posts describing violence and the perpetrators.

From the total 600 starting posts, 39% (95% CI: 35–43%) described violence (Figure 1). In these posts, the perpetrator was most frequently a former or current intimate partner (39%), followed by a close family member (18%)—father, mother, brother, sister, or son. The perpetrator could not be recognized in 13% of posts describing violence. Less frequently, the perpetrator was an acquaintance or extended family member. 

### 3.2. Motives for Visiting an Online Support Group (OSG)

**Figure 2:**
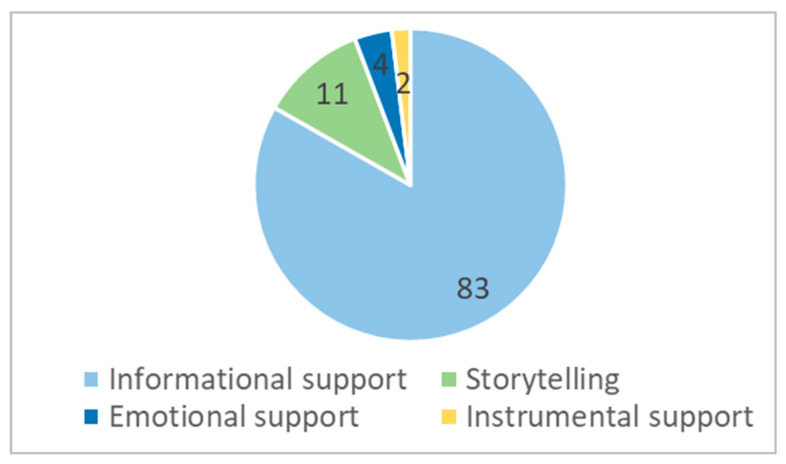
Motives for visiting the OSG.

The majority of starting posts in the online support group Women in Need included support seeking (Figure 2). The most common type of support the authors were looking for was informational (83%), sometimes in a very factual way, as shown by the following example:

*Dear all, I am in the 12th week of pregnancy and all indications show that my employment will end prematurely with no fault on my behalf (otherwise I have a fixed-term contract until February XXXX). Can this actually happen? Do I have any rights as a pregnant woman? Thanks, XXX*.

Support seekers were looking for advice, as many posts pertaining to informational support started with the following words:

*Hello. Maybe you can help me with some advice…*

or ended with the following:

*Can anybody offer me some advice?*

There were 4% of starting posts in which the authors were looking for emotional support. The need for such support was expressed by sentences such as:

*I’m just asking for a little encouragement because I don’t want to lose the opportunity by waiting indefinitely.*

or:

*What should I do, I need hugs and love, I need support, a kind word, especially during pregnancy. I need understanding.*

or:

*I don’t know what I want from you, maybe just some encouragement that everything will be alright.*

There were 11% of posts in which the authors were telling their story or experience of abuse that often ended with words of despair. An example of one such ending to a post is the following:

*When do you know it doesn’t work anymore? I don’t have suicidal thoughts, I would just turn off the program or fast forward it. Unfortunately, it doesn’t work. I don’t even know what I want to do with this message, I just know that I’m tired.*

Such posts with descriptions of difficult situations and/or the feelings accompanying them could also be viewed as a subtle way of seeking emotional support. A study on the techniques used by Facebook users to obtain emotional support revealed that one of them is rich storytelling [20].

The least common type of support that was asked for was instrumental support, found in just around 2% of posts. An example of one such post is the following:

*I am urgently looking for a room in XXX with a shared kitchen and bathroom, possibly in exchange for helping an elderly woman. I am a calm, tidy woman, a non-smoker without children. I would like to move in sometime in XXX. My phone number is: XXX XXX XXX. I hope that someone can help me or you know someone who would share an apartment with me.*

### 3.3. Topics of Online Discussions

There were 10 topics of discussion among members of the online community (Table 1). The most common topic, raised in 14% of posts, was a description of current psychological distress due to the circumstances or situation experienced by the author. Women described the strains in their relationships with their partners because of their absences and unwillingness to contribute financially or consent to a divorce. Women described their loneliness, partners’ infidelity, or other types of mistreatment, ranging from constant quarrelling or verbal abuse to physical assault.

Professional advice was offered in 13% of all posts. These posts were mainly written by the moderators, social workers, or others employed in safe houses. Building on their experience and professional knowledge, the moderators educated women about their possibilities, shed light on the situation the women were experiencing, and encouraged them to make the first step towards improving their lives. The latter included giving advice on going to a safe house or associations that help women in such situations. They thus tried to provide a viable solution to the problems women faced, while attempting to reduce their concerns by directly addressing their fears.

Another 13% of posts included reactions to different posts by offering advice (informational support), congratulations, empathy, sympathy, and encouragement (emotional support), as well as offering housing, toys, food, or clothes (instrumental support). This was conducted by other members of the online support group with similar experiences or by moderators.

There were two topics related to sexual abuse, each accounting for 12% of posts. Such posts described an actual experience of sexual abuse, often in childhood or adolescence, and its effects on the victims’ lives. They reported fears of visiting gynecologists, lack of sexual desire, or unwillingness to have a romantic relationship. There were also posts about the recognition of sexual abuse and possible support for victims.

A number of women turned to the OSG to find a solution regarding their dire financial situation (12% of posts). They wrote about their struggle to provide for themselves and their children, describing the unwillingness of their ex-partners to pay alimony or child support and asking for advice on how to cope with the resulting financial burden.

Topics that appeared in less than 10% of the posts included negative attitudes towards women from various sources, not necessarily against the author of the post but their friends, mothers, daughters, and so on (8% of posts), difficulties they were experiencing in their partnership, including infidelity and divorce (6%), possible coping mechanisms to solve the situations they faced, such as reading materials, therapy, or going to a safe house (6%), and legal advice or how to provide evidence of abuse to the police (4%). 

### 3.4. Nature of Social Support

**Figure 3:**
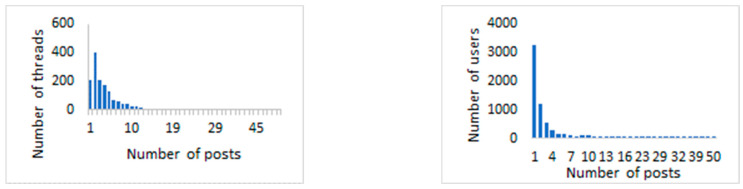
Number of posts per thread (**left**) and number of posts per user (**right**).

Most commonly, there were two posts per thread (see the left-hand side of Figure 3), while threads with a large number of posts were rare. The threads with the largest number of posts were those about alimony and child support (55 posts), sexual abuse—my story (56 posts), and family violence (70 posts). Almost half of the users (47%) coming to the online support community published a single post, 18% published two posts, 7% three posts, and 13% more than 10 posts (see right-hand side of Figure 3).

**Figure 4:**
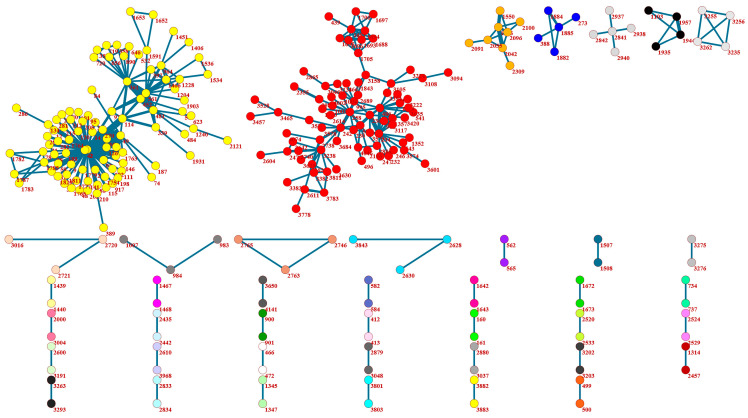
Network of users posting together in at least two threads.

Exploring the network of users that posted together in at least two threads showed that two larger supportive communities existed (Figure 4). In these communities, the users collaborated with others to a greater extent, thus forming supportive ties. Other groups included smaller numbers of members, with the most common being those with only two individuals. 

**Figure 5:**
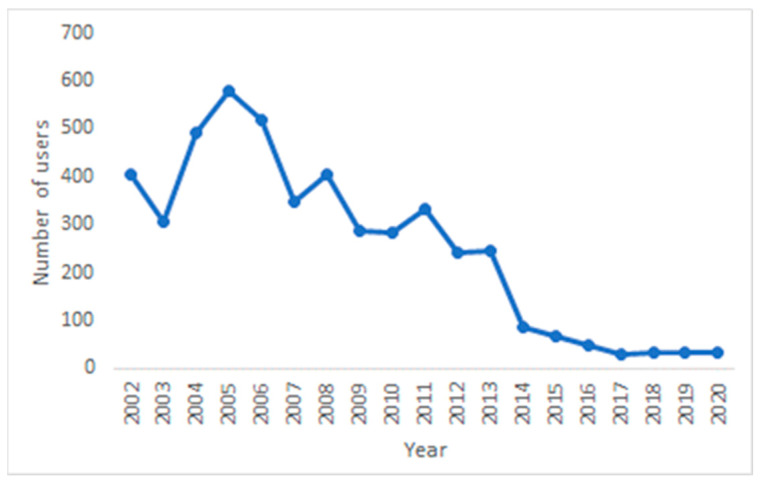
Number of users over time.

Figure 5 illustrates the number of OSG members per year in the period between 2002—when the OSG was formed—and the end of 2020. It is evident that, after 2013, there was a significant drop in the number of OSG members, and the number has not increased since.

## 4. Discussion

The first two research questions addressed the motives for visiting the online support community Women in Need and the share of posts including the description of violence. Manual annotation of 600 randomly selected starting posts revealed that 40% of these contained a description of violence, and the most prevalent was the description of IPV. The findings of the FRA [4] also confirm that this is the most common type of violence against women. The main motive for women to join the support group and ask a question was seeking support, with most looking for informational support. In this sense, the Women in Need OSG is similar to Q&A sites, as analyzed by Westbrook [18], where the participants were also mainly seeking informational support. After making their first post, about 50% of the women in the focal OSG group never returned to reply or start a new thread, possibly because they obtained the information they were looking for.

Looking for a supportive community was also one of the main motives for rape survivors when visiting a public rape survivor forum on Reddit [19]. They were, however, more often looking for emotional rather than informational support, which was also the case in a Chinese study analyzing the Twitter messages of victims of IPV [17]. In our study, only 4% of the 600 randomly chosen starting posts included emotional support seeking. However, if we consider storytelling as a technique for eliciting emotional support [20], then the share of starting posts seeking such support increases to 15%. 

Taking into account all the posts of the researched OSG, various types of social support were included in several topics. Informational, emotional, and instrumental support was offered by the social workers and psychologists working in safe houses (included in 13% of posts) and/or other members of the OSG (included an additional 13% of posts). The former included information about institutions and associations that help women who are victims of various kinds of violence, as well as offering reassurance and encouragement for women to take the first step towards escaping the violence and other difficult situations in which they found themselves. Such reassurance and encouragement was also found in the posts made by other members of the OSG, who urged others to take the necessary steps to escape their difficult situations, as well as provided empathy and sympathy to the support seekers. This finding is in line with that of Rempel et al. [14], which reported that online interventions can help victims to make the first move towards leaving their abusive partners. Tarzia et al. [16] also showed that many elements of offline social support can successfully be transferred online. The women in the OSG were also looking for referrals to psychologists, associations, and lawyers dealing with victims of violence and especially in intimate relationships (topics about legal advice and coping—10%) or advice on how to address their difficult financial situations (12%). In this sense, they were mainly looking for informational support. They were also coming to the OSG to share details of their psychological distress arising from the problematic relationships they had with their partners (in most cases, although, sometimes, the problem was with another person) and to tell their stories (14% of all posts). Storytelling was mostly present in those posts where the authors described their experiences of sexual abuse—usually many years before (12%)—as well as in those describing the psychological distress arising from their relationships (14%). If rich storytelling is a technique for obtaining emotional support, the need for this type of support was the most pronounced in the posts that were examined. The basis for effective social support in an online environment is the more engaged participation of users and a higher number of members who can provide novel information [8]. However, almost two thirds (65%) of the members posted no more than two messages to the group, and about half of them only posted one, which suggests that these women were rather passive in this regard and tended to quickly disengage from the online discussions. This obviously made continued monitoring impossible, as well as helping victims with advice on both short- and long-term actions. This thus suggests there is a relatively low possibility of offering useful social support to members of the investigated OSG, which is further supported by other findings of this research. One of them is the decreasing number of members over time, as this figure has been falling for some time and has been low for the last five years. Furthermore, most threads include only two posts, meaning no lively discussion among members developed. This finding was also supported by the results of the network analysis. There were only two large groups of intertwined members who developed relationships by providing and obtaining information and emotional support. Research shows that the more engaged members of an online community can benefit the most from online support [8,21], and the absence of such engagement is yet another indication that the social support available in the Women in Need OSG had difficulties meeting the users’ needs. While this was not necessarily true throughout the investigated period, it certainly was during the last five or six years. The analyzed OSG is thus in decline, possibly because of similar online communities developing on social media platforms, such as Twitter or Facebook, or because of (over)expansion of OSGs and services the site that hosts this OSG offers. The proactive engagement of different members is also important from the point of view of social reactions to a victim’s disclosure. Online, these tend to be positive, at least when a victim is describing sexual violence [22]. Positive reactions are also expected to a greater extent when a victim is looking for support from informal sources [5].

Perhaps the most surprising finding of our study is the women’s hesitation to engage in more in-depth online discussions. Since other members of the OSG, as well as the staff of safe houses who get involved, are adopting informal or less formal roles online, such a large drop in participation is unexpected. Our tentative interpretation includes the specific emotional and social characteristics of the group members, who predominantly have at least one (likely traumatic) experience of violence. Consequently, it might be that these women’s offline social functioning is weaker or impaired [23,24] and relating to others online may not be any different. At least some socializing skills, trust, and security must be present to engage and form a bond online, the same as offline. On the other hand, sometimes support is perceived and received on a much smaller scale than we would usually expect. Sometimes a feeling of validation alone might be enough [29], even though there is no direct interaction between members. Moreover, something as simple as a comment or a reaction to a post with an emoji can be felt as support [30].

Two major contributions of our study can be highlighted. Firstly, considering the sensitivity of the topic, the data were obtained with a noninvasive method. The users participated in online discussions willingly and anonymously. They freely discussed topics that they were interested in and expressed their concerns. Such natural discourses under the veil of anonymity can reflect users’ genuine opinions, experiences, and feelings. The analysis of such, spontaneously provided, content can result in more valid conclusions than when using more conventional research methods. Secondly, our findings give a good basis for establishing a Q&A page for “women in need”, where they could readily find the answers to the most pressing questions and issues. Our research showed that they mainly seek informational support. The analyzed online OSG as well as other similar sites could be improved by providing the incoming users with information that is most sought by users. These could include the list of safe houses along with links to their web pages and a short description of their main purpose and procedure of admission. There could also be links to other supportive communities, online and offline, a list, and links to social services associations that primarily help women experiencing domestic or other kinds of violence. Some names of psychologists and lawyers that would agree to engage as a sort of first responder could also be provided. Basic information regarding alimonies and legal rights (financial and related to childcare) a person has after a divorce or separation from a partner should be available. Such information could improve the functionality of online support group sites and turn around the negative trend of the number of new participants. 

There are also some limitations of our study. The drawback of the used noninvasive research approach is that the researcher cannot ask participants for further explanation or ask about additional context that is not spontaneously provided by the participant. As, due to the anonymity of participants, there are no demographic data provided, it is not possible to assess the sample representativeness. Consequently, the generalizability of findings to other women that do not participate in such online discussions is limited. 

## 5. Conclusions

Online interventions definitely have a lot of potential to help victims of IPV [16] and could be an effective channel for promoting anti-IPV campaigns [17]. However, they can only offer a certain level of help and support, as the process of leaving an abusive relationship is complex and includes many individual and subjective factors.

## Figures and Tables

**Table 1:** Topic size (% of posts), title, and key words.

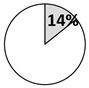	PSYCHOLOGICAL DISTRESS	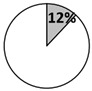	FINANCIAL HARDSHIP
	job, husband, father, child, strength		alimony, apartment, social, pay, help
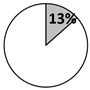	PROFESSIONAL ADVICE	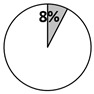	NEGATIVE ATTITUDES TOWARDS WOMEN
	life, relationship, love, partner, feeling		man, cry, boyfriend, happen, know
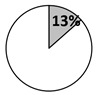	SOCIAL SUPPORT	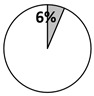	PARTNERSHIP
	violence, woman, safe house, help, leave		woman, life, help, parent, wish, time
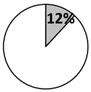	SEXUAL ABUSE—EXPERIENCE	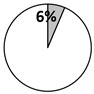	COPING
	abuse, knowing, sexual, childhood, fear		safe house, maternity, help, violence, consult
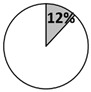	SEXUAL ABUSE—RECOGNITION AND HELP	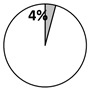	LEGAL ADVICE AND REPORTING TO THE POLICE
	abuse, sexual, perpetrator, victim, question		violence, court, police, social service, procedure

## Data Availability

Data can be obtained upon request from the authors upon approval of the med.over.net.
